# Fitness Costs of Maternal Ornaments and Prenatal Corticosterone Manifest as Reduced Offspring Survival and Sexual Ornament Expression

**DOI:** 10.3389/fendo.2022.801834

**Published:** 2022-03-03

**Authors:** Braulio A. Assis, Julian D. Avery, Ryan L. Earley, Tracy Langkilde

**Affiliations:** ^1^ Department of Biology, The Pennsylvania State University, University Park, PA, United States; ^2^ Intercollege Graduate Degree Program in Ecology, The Pennsylvania State University, University Park, PA, United States; ^3^ The Department of Ecosystem Science and Management, The Pennsylvania State University, University Park, PA, United States; ^4^ Department of Biological Sciences, University of Alabama, Tuscaloosa, AL, United States

**Keywords:** maternal effects, stress, color, heritability, female ornamentation, *Sceloporus*

## Abstract

Colorful traits (i.e., ornaments) that signal quality have well-established relationships with individual condition and physiology. Furthermore, ornaments expressed in females may have indirect fitness effects in offspring *via* the prenatal physiology associated with, and social consequences of, these signaling traits. Here we examine the influence of prenatal maternal physiology and phenotype on condition-dependent signals of their offspring in adulthood. Specifically, we explore how prenatal maternal testosterone, corticosterone, and ornament color and size correlate with female and male offspring survival to adulthood and ornament quality in the lizard *Sceloporus undulatus*. Offspring of females with more saturated badges and high prenatal corticosterone were less likely to survive to maturity. Badge saturation and area were negatively correlated between mothers and their male offspring, and uncorrelated to those in female offspring. Maternal prenatal corticosterone was correlated negatively with badge saturation of male offspring in adulthood. Our results indicate that maternal ornamentation and prenatal concentrations of a stress-relevant hormone can lead to compounding fitness costs by reducing offspring survival to maturity and impairing expression of a signal of quality in surviving males. This mechanism may occur in concert with social costs of ornamentation in mothers. Intergenerational effects of female ornamentation and prenatal stress may be interdependent drivers of balancing selection and intralocus sexual conflict over signaling traits.

## Introduction

Many animal species employ visually conspicuous traits in communication and signaling (1, 2). The expression and strength of such signals can be mediated by hormones, such as sex steroids (3) and glucocorticoids involved in stress responses (4). However, individual phenotype and fitness can also be influenced by maternal hormones during the prenatal phase (5). Evidence for maternal physiological effects on progeny are widespread and diverse, ranging from phenotypic expression (6) to hatching success and survival (7). In females, the prevalence and adaptive potential of sexual ornaments has received significant attention (8–12), but the maternal influence – either *via* genetic inheritance or prenatal effects – on adaptive expression of these traits in offspring remains unclear. Intergenerational associations between ornaments in offspring and maternal hormones and/or maternal inheritance might have important fitness consequences and could represent alternative ways in which female ornamentation can be evolutionarily relevant.

Maternal prenatal physiology can affect the fitness of progeny in various ways. A commonly proposed mechanism for these intergenerational responses is embryonic exposure *in ovo* or *in utero* to circulating maternal hormones that cross the amniotic barrier such as androgens (13, 14) and glucocorticoids (15). Recent work highlights the adaptive potential of prenatal effects within changing environments (16, 17), including behavioral and morphological changes in offspring that enhance survival (18). Maternal glucocorticoids may also impair offspring fitness by reducing their survival (19, 20) or egg hatching success (21). Thus, the generality of adaptive transgenerational effects is still debated (22, 23), and their range of influence requires further investigation. Condition-dependent signals such as sexual ornaments are often co-regulated by hormones with intergenerational potential (24), and thus constitute fitness-relevant traits that might be influenced by maternal effects.

**Figure 1:**
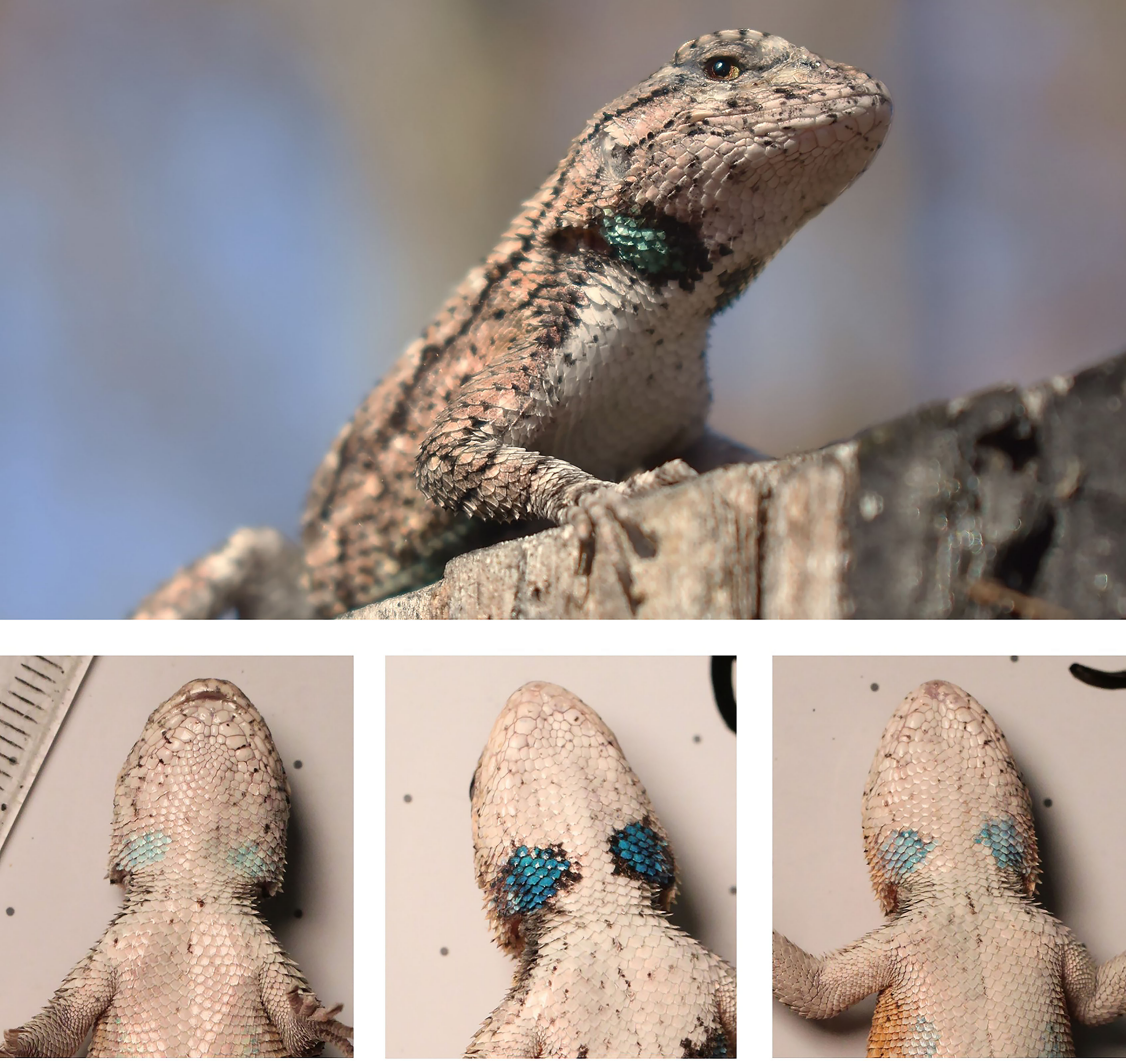
Above: eastern fence lizard in early adulthood in early adulthood and released after the conclusion of this study; below: representative photos of mature offspring used in this study. Left: female; center: male with highly saturated badges; right: male with low saturation badges. All lizards were approximately 323 days of age.

Many species of the lizard family Phrynosomatidae exhibit conspicuous ventral coloration that functions in intraspecific communication (25, 26). In the eastern fence lizard *Sceloporus undulatus*, sexually mature males bear blue ventral and gular badges formed by iridophores and melanophores (**Figure 1**). Males with more saturated badges have higher body condition and immune response, indicating that color is an advantageous signal of superior quality to conspecifics (27). Females have badges that lack the melanin pigmentation seen in males and consequently show a fainter (less saturated) blue (28). The fitness consequences of ornamentation in female fence lizards is still ambiguous, with studies linking it to costs (25, 29) and benefits (30). Further, the size and saturation of these color patches are co-regulated by individual condition and physiology, with testosterone (T), dihydrotestosterone (DHT), and corticosterone (CORT) playing important roles (27, 31, 32). If badges of fence lizards are correlated between mothers and offspring, or if prenatal maternal physiology is relevant to trait development in offspring, then ornamentation in mothers could be associated with increased grand-parentage because male offspring with more vivid badges are more successful in attracting mates (33).

We hypothesized that maternal T and CORT during pregnancy would affect clutch survival and ornament quality in offspring. We monitored 120 fence lizard hatchlings from 12 mothers throughout early ontogeny (i.e., hatching to sexual maturity), and predicted that: 1) high maternal CORT would reduce offspring survival to sexual maturity while low maternal CORT and high maternal T would improve offspring ornament quality (color saturation and badge area) due to known intraindividual relationships between these hormones and traits (27), and 2) maternal ornament quality would be correlated with that of their offspring due to maternal genetic inheritance. Support for these predictions could indicate indirect fitness benefits of female ornamentation *via* maternal investment in their offspring, with maternal physiology and genetic inheritance as potential mechanisms maintaining secondary sexual characteristics in females.

## Methods

### Animal Collection and Housing

We collected gravid females from the St. Francis National Forest and private lands in Lee County, Arkansas (AR), USA (n = 9), and from the Edgar Evins State Park and Land Between the Lakes National Recreation Area, Tennessee (TN), USA (n = 3). Immediately after capture, we collected blood samples from the post-orbital sinus (34) which were kept on ice until centrifuged for plasma extraction and storage at -20°C for future hormone assays. For all lizards, time to capture and obtain a blood sample was < 285 seconds (mean 192 seconds ± 47 SE). For one individual, the time elapsed from its sighting until blood sampling was 633 seconds. It has been demonstrated for this species that upregulation of CORT does not occur until 600 seconds after disturbance (35). Furthermore, this individual’s plasma CORT concentration was 13.24ng/mL, close the population mean for this study (15.41 ± 2.14 SE; range: 1.10, 31.03). These females were brought to an animal housing facility where they laid eggs, which were incubated at 30°C until hatching (47 females and 44 males from AR; 15 females and 14 males from TN). Sex of hatchlings was determined by the presence of post-anal scales in males. Up to five non-siblings from mothers of the same site were housed in common garden conditions, as described elsewhere (27). Thus, offspring were raised in environments distinct from their mothers’, allowing us to reduce confounding effects of shared environmental effects on badge development. At 321 days of age, snout-to-vent length for each offspring was close to or greater than 54 mm and they were thus considered sexually mature (36). At that point, we quantified badge color and area as performed in their mothers, described below.

### Hormone and Ornament Quantification

Maternal T and CORT were quantified using enzyme immunoassays (Cayman Chemical, Ann Arbor, MI). Protocols for plasma steroid extraction and immunoassays are detailed in the **Supplementary Material**.

We took ventral photographs of each offspring (at maturity) and their mothers (prior to laying) using uniform camera settings and lighting conditions, and with a metric ruler in view as a scale for badge measurements (**Figure 1**). We only quantified throat badges since they can occur in males and females and allow comparison between sexes, whereas abdominal badges are only rarely seen in females. Relative badge area was calculated as the residual of badge area linearly regressed on head area (37), both of which were measured using ImageJ (38).

Badge color was measured on the blue portion of each lizard’s left throat badge using an Ocean Optics Jaz UV/VIS spectrometer. Saturation was calculated as light reflectance at the range of peak wavelength reflectance ± 50 nm, divided by total light reflectance between 300 and 700 nm (39, 40). Fence lizard badges are responsive to temperature, becoming more saturated with increasing temperatures (40–42). We corrected color saturation for internal body temperature during spectrophotometric measurements by extracting the residuals of a linear regression of saturation on body temperature (measured in the cloaca using a Fluke 51/52 II 60 HZ thermocouple thermometer). Additional information about color measurement can be found in the **Supplementary Material**.

### Statistical Analysis

All statistical analyses were conducted using R v. 4.0.3 (43) and the packages *lme4* (44) and *lmerTest* (45) for linear and generalized linear mixed-effects models (LMM and GLMM, respectively). To verify model assumptions of no multicollinearity, predictors in all models were inspected for high variance inflation factors (VIF) using the *car* package (46). LMMs had no observations that were highly influential based on Cook’s Distance (all < 0.8).

To estimate the probability of offspring survival, we assigned a value of 1 to all hatchlings that were alive at 321 days of age, and a value of 0 to those that were not. We began by entering this binary code as the response variable in a logistic GLMM with the following predictors: 2-way interactions between hatchling sex and maternal T, maternal CORT, maternal badge saturation, and maternal badge area, as well as their individual main effects. To control for variation among mothers in hatching success of their offspring, we included the number of eggs hatched per clutch as a covariate. Maternal identity nested within her site of origin was included as a random effect. However, the maternal T x sex and maternal CORT x sex interactions had very high VIFs (72.9 and 28.5, respectively) and were thus removed from the model. All other VIFs < 2.9.

Intergenerational correlations for badge saturation and badge area were analyzed for male and female offspring separately. Thus, four LMM were built (for the two badge traits and the two offspring sexes), each with the following predictors: maternal badge saturation *or* area (for the model with the corresponding trait in offspring), maternal T, and maternal CORT. In addition, we included interaction terms between maternal badge traits and hormones (T or CORT), but none were significant and were thus removed from the models (for female offspring, all p > 0.24; for male offspring, all p > 0.845). Maternal identity nested within site of origin was included as a random effect.

To investigate heritability of badge traits we used univariate LMM for each badge trait (color saturation and area) of mothers and offspring, along with site and maternal identity as random effects. These were compared to identical models without the maternal identity effect using likelihood ratio tests based on a χ^2^ distribution. Significant differences between models would indicate that maternal identity explained a significant proportion of variation in offspring traits, providing evidence for heritability.

## Results

**Figure 2:**
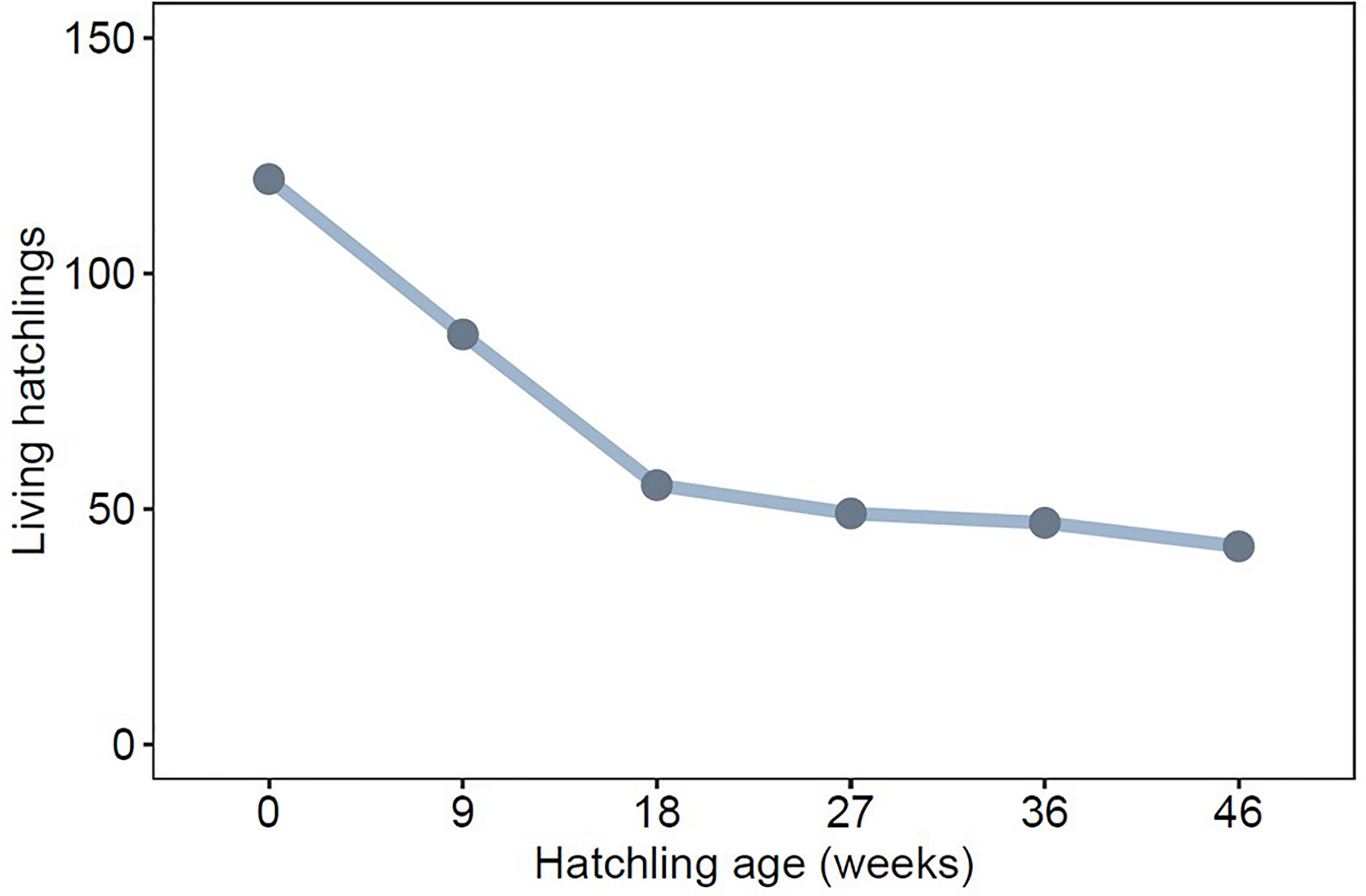
Total number of surviving lab-reared offspring across eleven clutches at weekly age intervals.

**Figure 3:**
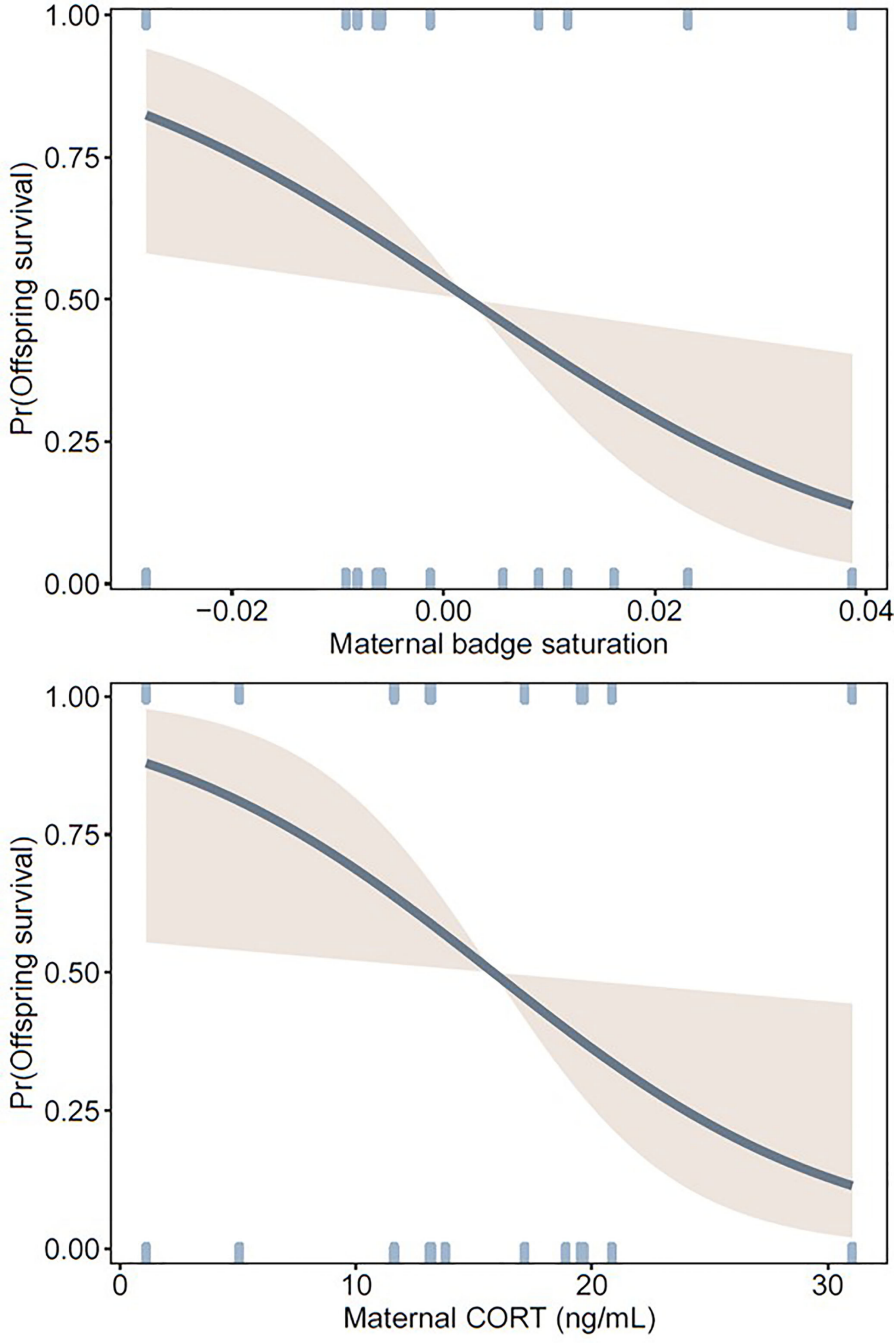
Probability curves showing offspring survival to 323 ± 2 days of age (at which they had reached sexual maturity) in relation to statistically significant predictors (maternal badge saturation and CORT) in a logistic regression. Other model parameters are shown in **Table 1**. Maternal badge saturation was corrected for body temperature at the time of measurement (see *Methods*).

Of 120 hatchlings, 42 survived to 321 days of age. Across all clutches, greatest mortality occurred before hatchlings reached 126 days (18 weeks) (83% of all mortality, **Figure 2**), which is well before sexual maturity. Offspring were more likely to survive to maturity when their mothers had less saturated badges and lower levels of baseline CORT during pregnancy (**Table 1**, **Figure 3**). Offspring sex was not a significant predictor of survival, indicating that mortality rate was not skewed towards either sex (**Table 1**).

**Table 1:** Logistic regression output for the probability of offspring survival to 323 ± 2 days of age, at which they have reached sexual maturity.

Offspring survival	β	SE	z	P
Intercept	-2.75	1.72	-1.59	0.111
Offspring sex	-0.22	0.42	-0.51	0.611
**Maternal badge saturation**	**-50.61**	**20.44**	**-2.48**	**0.013**
Maternal badge area	-0.30	15.59	-0.02	0.984
Maternal T	2.09	1.16	1.81	0.070
**Maternal CORT**	**-0.13**	**0.06**	**-2.20**	**0.028**
# eggs hatched	0.12	0.09	1.45	0.147
Maternal badge saturation * Offspring sex	23.21	24.85	0.93	0.348
Maternal badge area * Offspring sex	29.88	18.93	1.58	0.114

Maternal badge traits and hormone concentrations were measured during pregnancy. Maternal identity and site of origin included as random effects. Predictor variables in bold are statistically significant.

**Figure 4:**
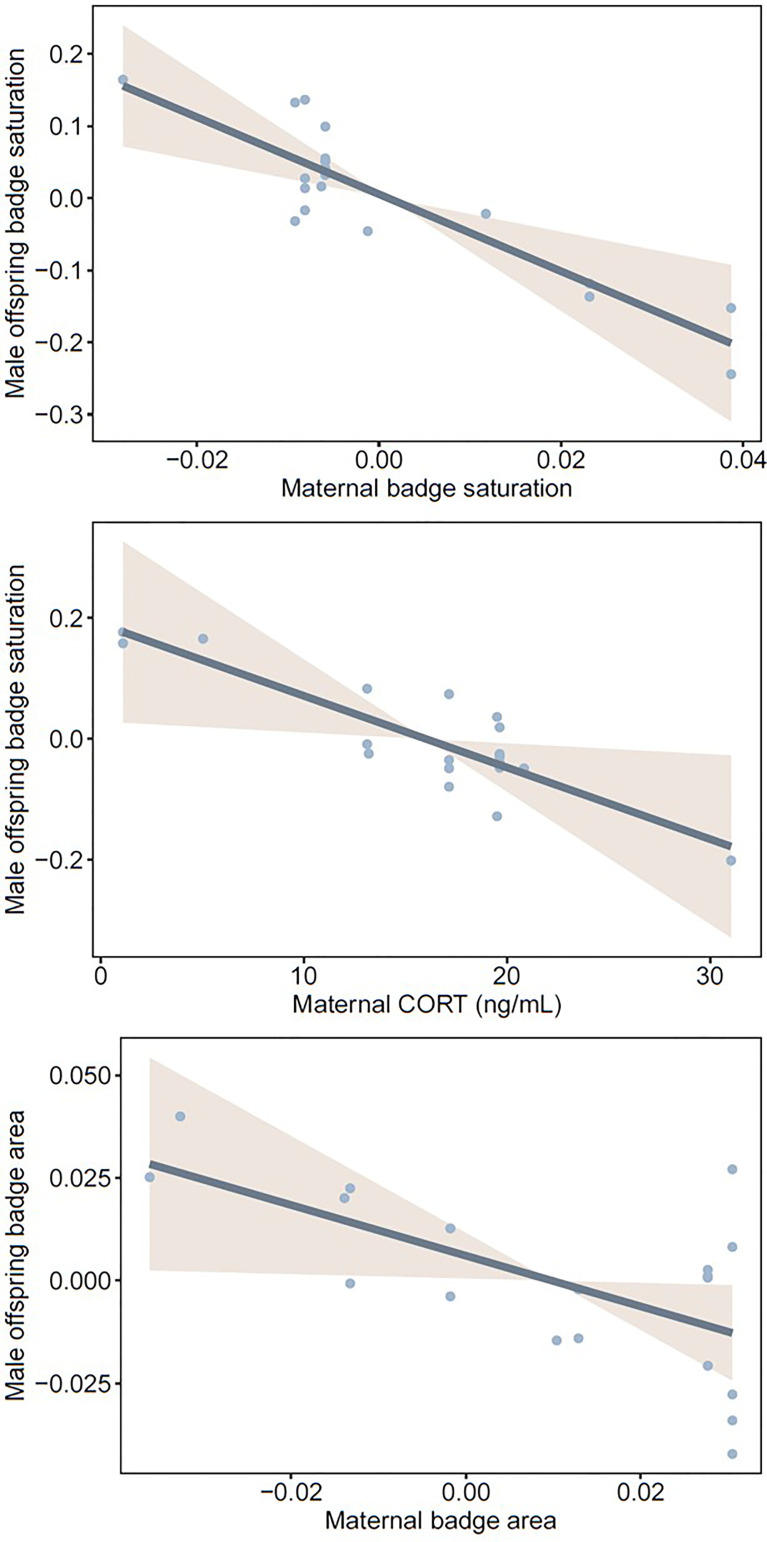
Statistically significant relationships between male offspring badge quality (saturation and area) and either maternal badge quality (saturation and area) or maternal baseline CORT during pregnancy. Badge saturation was corrected for body temperature at the time of measurement, and badge area was quantified in relation to head area (see *Methods*).

Badge saturation in mature male offspring was higher if their mothers had less saturated badges and lower baseline CORT (**Table 2a**, **Figure 4**). Badge area in mature male offspring was larger if their mothers had smaller badges, but was not associated with maternal CORT (**Table 2b**, **Figure 4**). For female offspring, the two badge traits were not associated with maternal badge characteristics or prenatal maternal T or CORT (**Tables 2c, d** and **Figure S1**). Of note, maternal T approached statistical significance as a predictor of badge saturation and badge area in male (p = 0.099 and p = 0.071, respectively), but not female offspring; mothers with higher T tended to have male offspring with larger, more saturated badges. Likelihood ratio tests indicated that models with and without maternal identity were equivalent in explaining variation in offspring badge traits (all p > 0.16), thus providing no evidence for heritability of color saturation or area in offspring.

**Table 2:** Output of four linear mixed-effects models for two measures of ornament quality (badge color saturation and badge area) in male and female offspring and their relationships with maternal phenotype and endocrine status during pregnancy.

a	Male badge saturation	β	SE	t	P
	Intercept	-0.07	0.09	-0.76	0.461
	**Maternal badge saturation**	**-5.34**	**1.47**	**-3.63**	**0.003**
	Maternal T	0.15	0.09	1.77	0.099
	**Maternal CORT**	**-0.01**	**0.005**	**-2.31**	**0.036**
b	Male badge area	β	SE	t	P
	Intercept	-0.06	0.03	-1.92	0.075
	**Maternal badge area**	**-0.62**	**0.29**	**-2.15**	**0.049**
	Maternal T	0.05	0.03	1.94	0.071
	Maternal CORT	0	0	-0.81	0.432
c	Female badge saturation	β	SE	t	P
	Intercept	-0.02	0.02	-0.72	0.488
	Maternal badge saturation	-0.23	0.43	-0.54	0.605
	Maternal T	0.02	0.02	1.04	0.320
	Maternal CORT	0	0	-0.69	0.504
d	Female badge area	β	SE	t	P
	Intercept	-0.06	0.03	-1.85	0.104
	Maternal badge area	-0.24	0.36	-0.68	0.527
	Maternal T	0.04	0.02	1.63	0.145
	Maternal CORT	0	0	0.51	0.634

Maternal identity and site of origin were included as random effects. Predictor variables in bold are statistically significant.

## Discussion

Maternal physiology during pregnancy can have strong intergenerational fitness consequences by influencing offspring phenotypic expression (e.g., behavior, secondary sexual traits) and survival (5, 16). In parallel, signaling traits involved in social communication are often condition-dependent and have important fitness outcomes (47). However, studies that assess the contribution of maternal physiology and maternal inheritance to the expression of condition-dependent signals in offspring, and consequently to their fitness, are largely lacking. Our results show that naturally higher baseline levels of the stress-relevant hormone CORT along with more saturated badges in gravid female lizards were associated with a reduced probability that offspring would survive to sexual maturity. In surviving offspring, we observed sex-specific relationships: males with more saturated badges at maturity were born to mothers with lower badge saturation and lower CORT, and males with larger badges at maturity were born to mothers with smaller badges. The lack of evidence for genetic inheritance of this trait corroborates previous findings that their expression is condition-dependent (27). However, the negative relationship between maternal-offspring badge quality along with the negative effect of maternal prenatal CORT indicates a more complex developmental scenario. These effects could potentially involve trade-offs between ornament production and gamete production in females (12, 48). Non-exclusively, maternal life-history costs associated with the expression of a male-typical trait by females could also occur (25, 49), which we elaborate on below.

Negative effects of experimentally elevated maternal CORT on offspring survival have been previously documented in this species (21). Our results show that this effect holds for unmanipulated CORT levels in field-caught gravid female fence lizards, as females with naturally higher CORT levels had offspring with lower survival. After controlling for the effects of maternal T and ornament quality, number of eggs that successfully hatched within a clutch, and offspring sex, our model showed that maternal CORT explained a difference of ~75% in the probability of offspring surviving to adulthood (**Figure 2**). Although this effect was strong, limitations in the sampling regime along with the correlative nature of the analyses may reduce the generality of these observations. CORT is known to fluctuate throughout reproductive stages (50), and the snapshot nature of prenatal measurements may introduce error. Still, hatchlings in this study were protected from sources of mortality that would occur in natural environments (e.g., predation, resource availability, parasitism). Eliminating these strong drivers of mortality may help explain why maternal factors strongly predicted offspring survival. Future field studies incorporating natural drivers of mortality along with a more comprehensive prenatal hormonal profile (e.g., temporal sampling) would provide more insights into the fitness impacts of prenatal maternal physiology in ecologically relevant contexts.

Ornament quality in mothers (badge color saturation and area) was negatively associated with those of male, but not female, offspring (**Figures 4** and **S1**). Badges appear to be relevant for fitness in male fence lizards given their role in intraspecific agonistic displays (51; but see 52) and female mate choice (33), and given their potential to signal condition (27). Even though housing conditions were uniform in our experiments, juvenile lizards shared their environments with four to five non-siblings. It is possible that poorly ornamented male offspring of highly ornamented mothers incurred fitness costs due to social subordination, such as reduced opportunity to feed. This could drive an indirect negative relationship between high quality maternal ornamentation and low male offspring survival. Still, the mechanisms driving the negative relationship between ornament quality in mothers and their male offspring in this study are less clear. Although we found no evidence for maternal genetic inheritance of badges, the negative relationship between mother and male offspring badge quality could be driven by paternal traits. Ornamentation in both parents has been demonstrated to influence offspring fitness in common whitefish (53). We were unable to identify paternity of these clutches, or whether clutches had single or multiple sires, but previous associations between ornamentation and mate preference in this species can provide some insight. In laboratory settings, male fence lizards spend less time interacting with ornamented females during the mating season (29), and it has been suggested that males with smaller or fainter badges are less likely to be successful in social competition (33, 37). If these patterns hold true in natural settings, and if badge quality is genetically inherited paternally, then it should follow that the females with more vivid badges in our study most likely mated with males with poor badges, being also expressed in their male offspring. Future work on potential genetic contributions to fence lizard ornamentation will be necessary to explore this hypothesis.

More saturated maternal badges and elevated CORT during pregnancy predicted reduced survival to sexual maturity and reduced badge quality in adult male offspring, suggesting strong maternal effects on the fitness of male *S. undulatus*. Social interactions mediated by this condition-signaling trait – such as competition for food or dominance hierarchy formation within housing groups – could explain differences in survival to adulthood (but see 54). Even though baseline CORT and badge areas are negatively correlated within sexually mature individuals (27), our models also indicate significant maternal effects for each of those factors on offspring survival and male phenotype. Mechanisms underlying these effects remain speculative, but the role of CORT in modulating immune responses (34, 55) and energy metabolism (56), as well as the negative effect of CORT on yolk protein content in fence lizards (15), could be potential drivers of impaired embryonic development accompanied by reduced expression of a condition dependent signal (27) and high mortality.

In this study, we demonstrated that integration of maternal physiology and a socially relevant secondary sexual characteristic may reveal important intergenerational fitness consequences. Testing these relationships under natural field conditions where offspring might also experience food limitation or high predation risk, as well as integrating paternal effects, would shed important light on this process. If field observations support our findings, these relationships could indicate a scenario of balancing selection on a male secondary sexual trait driven by intralocus sexual conflict (57, 58). That is, the manifold costs (e.g., reproductive, social, physiological) of female expression of a sexual ornament prevents directional selection of this trait in males. The relationship between prenatal maternal physiology (e.g., CORT levels) and offspring survival, as well as offspring ornament development, might represent compounding fitness costs driving balancing selection *via* intralocus sexual conflict, thus precluding the evolution of complete sexual dimorphism.

## Data Availability Statement

The datasets presented in this study can be found in online repositories. The names of the repository/repositories and accession number(s) can be found below: ScholarSphere; https://doi.org/10.26207/fzq1-9k80.

## Ethics Statement

The animal study was reviewed and approved by The Pennsylvania State University’s Institutional Animal Care and Use Committee (IACUC).

## Author Contributions

Designed the study: BA and TL. Collected and analyzed the data: BA, JA, RE, and TL. Prepared the manuscript: BA, JA, RE, and TL. All authors contributed to the article and approved the submitted version.

## Funding

Funding was provided in part by the National Science Foundation (IOS- 1456655 to TL).

## Conflict of Interest

The authors declare that the research was conducted in the absence of any commercial or financial relationships that could be construed as a potential conflict of interest.

## Publisher’s Note

All claims expressed in this article are solely those of the authors and do not necessarily represent those of their affiliated organizations, or those of the publisher, the editors and the reviewers. Any product that may be evaluated in this article, or claim that may be made by its manufacturer, is not guaranteed or endorsed by the publisher.
