# Supplementary material for: Fitness Costs of Maternal Ornaments and Prenatal Corticosterone Manifest as Reduced Offspring Survival and Sexual Ornament Expression
Source: Front Endocrinol (Lausanne). 2022 Mar 3;13:801834. doi: 10.3389/fendo.2022.801834 (PMC8928773; doi:10.3389/fendo.2022.801834)
Supplement: Supplementary file 1 [file DataSheet_1.docx]

**Supplemental Materials**

*Husbandry*

After hatching, lizards were housed in groups of no more than five non-siblings in plastic containers (45 × 30 × 25 cm) furnished with a paper towel floor lining and a 15 × 15 cm piece of opaque corrugated plastic to be used as shelter, and lamps with a 60-W incandescent bulb (to allow thermoregulation) and a 13-watt UVB light bulb (ReptiGlo 10.0 Compact, ExoTerra), both suspended 25 cm above one end of the container and turned on daily from 08:00 to 16:00 h. Three days per week, lizards were fed *Acheta domesticus* crickets dusted with Zoo Med supplements: Reptivite with D3 twice a week, and Repti Calcium without D3 once a week (Zoo Med Laboratories Inc., San Luis Obispo, CA, USA). Water was provided *ad libitum*. The room was maintained at a temperature of 23°C and a 12:12 light cycle was provided by overhead lights.

*Color quantification*

The light source was emitted perpendicular to the subject and spectra were calculated relative to a diffuse white standard (Ocean Optics WS-1) using SpectraSuite (2006). We measured reflectance of the blue portion of the lizard’s left throat badge, with an integration time of 40 µs and a trigger period of 10 µs. We measured each lizard’s badge color three times, removing and replacing the probe each time within the blue region. For each measurement, SpectraSuite averaged five scans. We then used the R package pavo (Maia, Gruson, Endler, & White, 2019) to interpolate each spectrum to 1 nm intervals and used the procspec function to smooth the spectra with a span of 2/3. Afterwards, the three spectra for each individual were averaged resulting in a single spectrum for each individual lizard.

The hue of badges in fence lizards is sensitive to temperature (Assis, Jarrett, Koscky, Langkilde, & Avery, 2020; Langkilde & Boronow, 2010; Stephenson, Ihász, Byrd, Swierk, & Swierk, 2017), and for this reason we calculated saturation at a fluctuating spectral range that tracked the expressed hue. Therefore, saturation was determined as the ratio between brightness at the wavelength of maximal reflectance ± 50 nm, and total brightness from 300 to 700 nm. Our final color score for each individual consisted of the residuals of saturation linearly regressed on its body temperature at the time of measurement. Cloacal temperature was measured using a Fluke 51/52 II 60 HZ thermocouple thermometer (Fluke Corporation, Everett, WA, USA).

Individuals were placed on their backs and had two photographs taken from directly above, with a plastic ruler in the frame as a scale. The areas of the right throat badges were measured in ImageJ (Schneider, Rasband, & Eliceiri, 2012) using the Polygon tool, averaged from two photos per individual. The same procedure was used to measure the head area of all lizards, and our final measurement of badge area was calculated relative to head size by extracting the residuals of a linear regression of averaged badge area on averaged head area. Because both variables were two-dimensional, we did not square-root transform either area measurement.

*Hormone quantification*

Whole blood samples were centrifuged at 3,000 rcf for five minutes and plasma was pipetted into clean 0.2 mL microcentrifuge tubes for storage at -20°C. After thawing, 2 to 5 µL of plasma and 16 mL of Ultrapure water were combined in 18 x 150 mm borosilicate vials for steroid hormone extraction. Under vacuum pressure, samples were pulled over Thermo Scientific Hypersep C18 cartridges (3 cc, 500 mg bed weight) using high purity tubing (Tygon, Formulation 2275). Cartridges were initially primed with 2 x 2 mL of HPLC-grade methanol followed by 2 x 2 mL of distilled water, and after sample extraction, washed with 2 mL of distilled water. The free (unconjugated) hormone fraction was eluted from the cartridges using 2 x 2 mL of ethyl acetate and collected in 13 x 100 mm borosilicate vials. Ethyl acetate was evaporated from the samples by placing the vials under a light stream of high purity nitrogen gas in a 37°C water bath, and under an evaporating manifold. Hormone was resuspended in a solution of 5% ethanol and 95% enzyme-immunoassay (EIA) buffer (provided in the assay kit from Cayman Chemical). Plasma samples were reconstituted to a total volume of 350 µL. Suspensions were held overnight at 4°C until assay.

Cayman Chemical (Ann Arbor, MI) EIA kits were used to quantify T and CORT following manufacturer instructions (Catalog # 582701 and 501320, respectively). A pool of hormone was generated for each sample type (i.e., age groups) by combining 20 µL from each experimental sample. 300 µL of each pool was then serially diluted (1:1 to 1:128) to identify the appropriate sample dilution and to validate parallelism with the standard curve. Serial dilution curves were parallel with the standard curves in all cases (slope comparisons test, (Zar, 1996); T: t_12_ < 0.13, P > 0.90; CORT: t_12_ < 0.83, P > 0.43 ). All samples were assayed in duplicate.


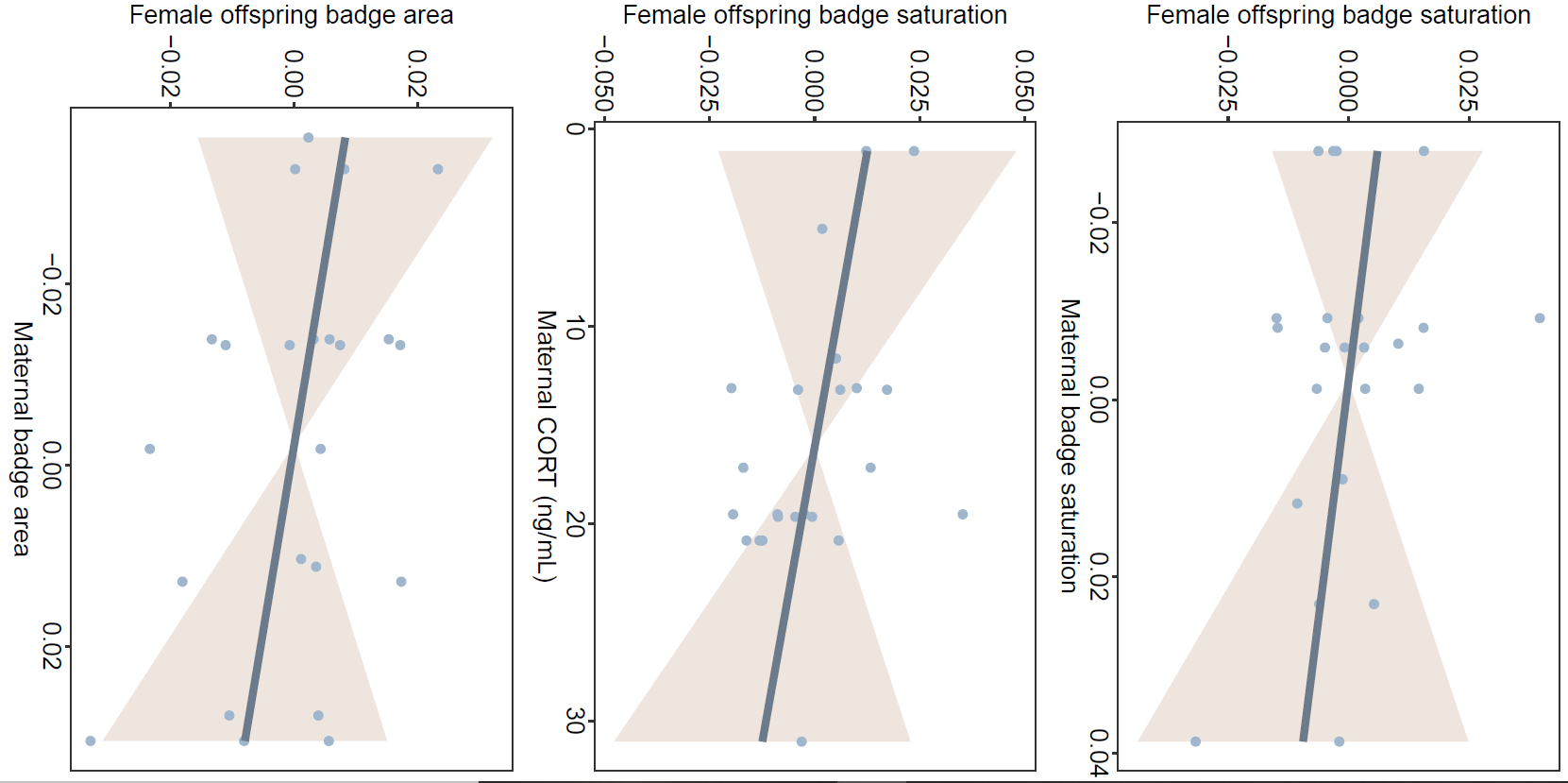


Figure S1: Nonsignificant relationships between female offspring badge quality (saturation and area) and maternal badge quality and baseline corticosterone. Badge saturation was corrected for body temperature at the time of measurement, and badge area was quantified in relation to head area (see *Methods*).

**References**

Assis, B. A., Jarrett, B. J. M., Koscky, G., Langkilde, T., & Avery, J. D. (2020). Plastic sexual ornaments: assessing temperature effects on color metrics in a color-changing reptile. *PLoS ONE*, *15*(5), e0233221. https://doi.org/10.1371/journal.pone.0233221

Langkilde, T., & Boronow, K. E. (2010). Color as a signal: the relationship between coloration and morphology in male eastern fence lizards, *Sceloporus undulatus*. *Journal of Herpetology*, *44*(2), 261–271. https://doi.org/10.1670/08-275.1

Maia, R., Gruson, H., Endler, J. A., & White, T. E. (2019). pavo 2: new tools for the spectral and spatial analysis of colour in r. *Methods in Ecology and Evolution*, *10*(7), 1097–1107. https://doi.org/10.1111/2041-210X.13174

Schneider, C. A., Rasband, W. S., & Eliceiri, K. W. (2012, July). NIH Image to ImageJ: 25 years of image analysis. *Nature Methods*, Vol. 9, pp. 671–675. https://doi.org/10.1038/nmeth.2089

Stephenson, B. P., Ihász, N., Byrd, D. C., Swierk, J., & Swierk, L. (2017). Temperature-dependent colour change is a function of sex and directionality of temperature shift in the eastern fence lizard (*Sceloporus undulatus*). *Biological Journal of the Linnean Society*, *120*(2), 396–409. https://doi.org/10.1111/bij.12870

Zar, H. J. (1996). *Biostatistical Analysis* (Third edit). Upper Saddle River: Prentice Hall.
